# Over a decade of single-center experience with thoracoscopic sympathicolysis for primary palmar hyperhidrosis: a case series

**DOI:** 10.1007/s00464-020-07769-0

**Published:** 2020-07-08

**Authors:** Adam Mol, Oliver J. Muensterer

**Affiliations:** 1 Department of Pediatric Surgery, Kraków, Poland; 2 Department of Pediatric Surgery, University Medical Center of the Johannes Gutenberg University Mainz, Langenbeckstrasse 1, 55131 Mainz, Germany

**Keywords:** Palmar hyperhidrosis, Thoracoscopic sympathicolysis, Compensatory sweating, Gustatory sweating, Horner syndrome

## Abstract

**Background:**

Primary palmar hyperhidrosis is a severely debilitating condition that can affect patients of any age. We report our experience with thoracoscopic sympathicolysis in a large cohort of children less than 14 years of age.

**Methods:**

All children who underwent thoracoscopic sympathicolysis from April 2005 through January 2017 were evaluated retrospectively. The procedure entailed bilateral bipolar fulguration of the second and third thoracic ganglia with transverse disruption of collateral nerve fibers along the third and fourth rib. Demographic information, as well as postoperative outcome, complications, and satisfaction were analyzed.

**Results:**

Over the 12 year study interval, a total of 102 children underwent thoracoscopic sympathicolysis for palmar hyperhidrosis. Complete follow-up was available for 98 patients (median age 12 [range 5–14] years; 38 boys [39%]). Median follow-up was 4 [range 2–12] years. Complete palmar dryness was achieved in 93 (95%) cases. One patient suffered postoperative unilateral ptosis, 6 reported gustatory sweating, and 65 experienced compensatory sweating. Average postoperative rating on a 1 (lowest) to 10 (highest) rating scale was 9, with 97 (99%) patients saying that they would undergo the procedure again.

**Conclusion:**

Our technique of thoracoscopic sympathicolysis in children was associated with very high postoperative satisfaction, despite a high rate of compensatory sweating and occasional autonomic gustatory sweating. Other more severe complications in this age group were rare.

Primary palmar hyperhidrosis is a disease of the autonomous nervous system characterized by excessive sweating over 6 months in duration that impairs daily activities [1]. In children, this can lead to handwriting difficulties in school, since the paper becomes soaked, causing the ink to run, compromising neatness and legibility. It also can cause social isolation and difficulty playing sports because a ball or other equipment may slip when gripped with a wet hand. The impact of palmar hyperhidrosis in children is often underappreciated. Patients are often ridiculed and become frustrated with their condition. As in adults, constantly having wet hands can stigmatize children and lead to introversion and low self-esteem.

Treatment options include topical therapy with aluminum salts (deodorant), oral anticholinergics such as glycopyrrolate or oxybutinin, calcium-channel blockers, clonidine, as well as iontophoresis, or botulinum injections [2]. Iontophoresis can improve palmar hyperhidrosis in up to 80% of cases but requires a series of treatments [3]. Oxybutinin has been reported to improve symptoms in 90% of cases, but requires long-term medication causing oropharyngeal dryness [4]. Finally, botulinum injections last only on average 7 months before symptoms return [5]. The only long-term therapy for severe palmar hyperhidrosis is operative sympathectomy or sympathicolysis, which is most often performed thoracoscopically.

We report our experience over more than a decade with thoracoscopic sympathicolysis using a special technique that entails bipolar fulguration of the second and third thoracic ganglia and interruption of the collateral fibers along the third and fourth ribs, and compare our outcome to that of other published series.

## Methods

### Study design

Approval was granted by the Ethics committee of the Institution (Y0941622T). The study was registered at www.researchregistry.com (researchregistry3903). All patients and their parents consented to participation of the study.

A retrospective review was performed on all patients 14 years of age and younger who underwent thoracoscopic sympathicolysis in the described technique from April 2005 through January 2017 at the University Hospital Las Palmas de Gran Canaria, Spain. Data were collected through paper and electronic medical records. A standardized schedule for follow-up for at least 2 years after the procedure was shared with the patients postoperatively and followed through by telephone or mail follow-up. Phone numbers were made available for any questions or complications, should they arise in the interim. All patients underwent a detailed preoperative and postoperative physical exam, and were asked to answer a postoperative questionnaire that included a satisfaction scale from 1 (least) to 10 (highest) satisfaction, along with a query of postoperative symptoms and complications.

### Surgical technique (Fig. 1)

**Fig. 1:**
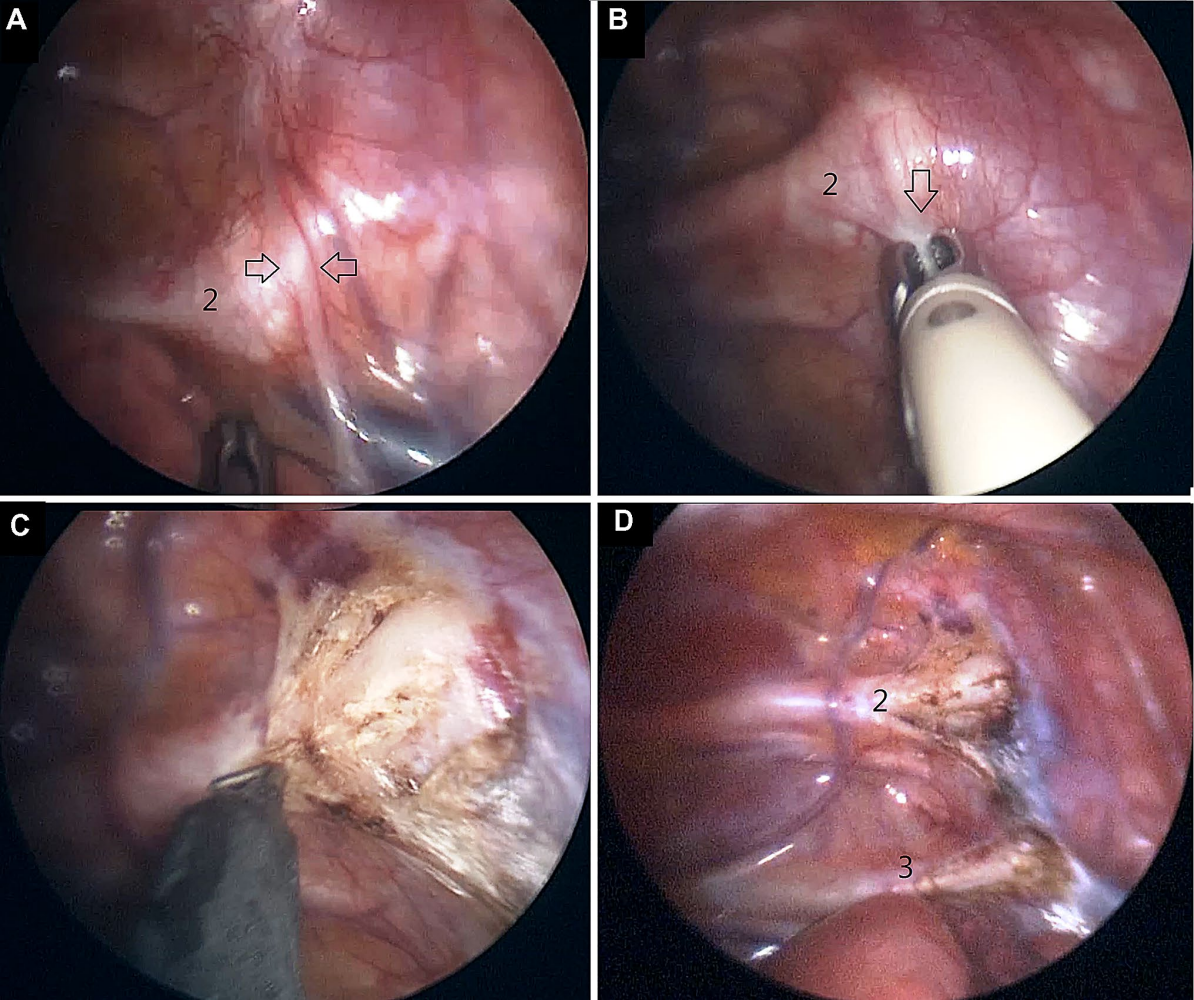
Technique of thoracoscopic sympathicolysis. The sympathetic chain (between arrows) is identified over the head of the second rib (2) as seen in (**A**). It is fulgurated using bipolar diathermy through the intact pleura (**B**, arrow). Using electrocautery hook, the pleura and periosteum is opened laterally for 3 cm on top of the rib (**C**), and the procedure is repeated along the 3rd rib (**D**, 3)

Thoracoscopic sympathicolysis is performed under general anesthesia with conventional single-lumen intubation using low-pressure carbon dioxide insufflation (between 5 and a maximum of 10 mmHg). A one-time bilateral procedure is performed during the same anesthesia. All patients are placed in semi-sitting position with 90° abduction of the arms and the elbows slightly flexed. Two 5 mm incisions are made after the skin is injected with local anesthetic: the first in the mid axillary line in the third intercostal space, the second slightly anterior in the fifth intercostal space. The first trocar was placed using an open technique in apnea ventilation mode to minimize the risk of lung injury. Standard tidal volume ventilation is used by anesthesia during the procedure. The ipsilateral lung is collapsed by using valved trocars and insufflating carbon dioxide at a pressure of 1 mm Mercury over positive end expiratory pressure (PEEP). After identifying the sympathetic chain by vision and palpation, bipolar electrofulguration of the chain is performed through the intact pleura, starting just below the second rib (T2) and continuing down to the head of the fourth rib (T3). In doing so, the sympathetic chain “tenses and draws” itself below the intact parietal pleura, making the chain identification easier. Short single impulses are used, avoiding the dispersion of heat to adjacent structures. We use a 12 W energy setting in all cases, which is comparably low but proved adequate for effective fulguration of the sympathetic chain. Subsequently, a monopolar hook is used to make 3 cm long transverse incisions of the parietal pleura and periosteum along the third and fourth rib from medial to lateral, transecting the sympathetic chain and any collateral nerve fibers. After evacuation of the capnothorax, the trocars are removed and the wounds closed. No chest drains were left in place. All patients were operated by two board-certified pediatric surgeons.

## Results

### Demographics

During the study interval, a total of 102 patients were operated in the described fashion. Of those, complete follow-up was available from 98 patients. The median age of these at the time of the intervention was 12 years (Quartile 1: 11 years, Quartile 3: 14 years, range 5–14 years) and most were girls (60 girls vs 38 boys). Symptoms started at a mean age of 4.8 years, and parents of 23 patients said that the palmar hyperhidrosis was perceptible from infancy. The median follow-up was 4 years (range 2–12 years) after the procedure.

### Intraoperative and postoperative complications

Mean operative time was 90 min (95% confidence interval 85–95 min). Complete dryness of the hands was achieved in 93 (95%) patients, one hand in one patient stayed humid after an unsuccessful redo procedure, partial moistness was observed in 4 (4.1%) cases. A redo procedure was performed in 3 (3%) cases, 2 of which were successful. There were no intraoperative complications. Postoperatively, one patient presented with unilateral mild ptosis, which was not functionally impairing.

### Postoperative compensatory and gustatory sweating

Compensatory sweating (CS) appeared in 65 (66%) patients, 6 of which were transient and resolved spontaneously, another 12 reported decreasing CS with time. Long-term CS was therefore found in 59 (60%). Of those with any CS, 26 (44%) were not bothered by it at all, while only 3 saw it as a major impairment. The vast majority (64%) of those with CS reported CS within the first 3 months with a peak in the second month after the procedure. The location of CS were the feet in about half of patients 33), the back in 27, the trunk in 21, the axilla in 10, and the entire body in 5 patients. Autonomic gustatory sweating (upon tasting or smelling food) was reported in 6 patients.

### Satisfaction

Postoperative patient-rated satisfaction on a scale of 1–10, stratified by gender and compensatory sweating, is presented in Table 1. Overall, patients were very satisfied at a median of 9 (range 5–10). Boys with compensatory sweating were less satisfied than others (*p *= 0.03), while there was no difference in girls. Upon questioning, 97 patients said they would have the procedure performed again, only 1 patient did not (an adolescent with compensatory sweating).

**Table 1:** Satisfaction ratings on a scale of 1 (least) to 10 (highest) according to gender and postoperative compensatory sweating

Compensatory sweating (CS)	Boys (*n* = 38)		Girls (60)		All (98)	
[*n*]	*n*	Rating (Q1/Q3, range)	*n*	Rating (Q1/Q3, range)	*n*	Rating (Q1/Q3, range)
CS Yes [65]	27	9 (8, 10, 5–10)*	38	10 (9, 10, 6–10)	65	9 (9, 10, 5–10)
CS No [33]	11	10 (10, 10, 8–10)	22	10 (10, 10, 9–10)	33	10 (10, 10, 8–10)

*CS* compensatory sweating, Q1/Q3-first quartile/third quartile

*p = 0.032 by Mann–Whitney Test

## Discussion

Primary palmar hyperhidrosis (PPH) is a poorly understood derangement of the sympathetic system that leads to uncontrollable sweating of the hands. The overall incidence has been reported at around 5–15% of the population [6–8]. While PPH is well recognized in adults as a debilitating condition for social interaction, it has only received attention in children in the last two decades. Around 1.6% of adolescents and 0.6% of prepubertal children reportedly affected [9].

A literature search was performed using the subject terms syntax  〈thoracoscopic 〉 ,  〈sympathectomy〉 OR  〈sympathicolytic〉 ,  〈palmar hyperhidrosis〉,  〈children〉 in PubMed. The results were screened for relevance and the references in those respective studies were snowballed for a comprehensive overview of the subject. The results of all relevant studies were combined in a table to compare reported demographics and outcome. Table 2 shows the results of our systematic review. A total of 17 reviews were found, published from 1994 until 2019, all of them case series that included between 12 and 350 patients. There were no controlled trials or comparative studies. The techniques employed showed broad heterogeneity, as did the age ranges. Compensatory sweating was reported in 0–90%, patients stated that they would have the procedure performed again in 87–100%, while satisfaction rate in the published studies ranged from 57–100%.

**Table 2:** Systematic review of published case series of thoracoscopic sympathectomy or sympathicolysis in children

Author, year	Journal	Level (R rib, T thoracic vertebral)	Patients	Age range (y)	Mean age (y)	CS (%)	OP again (%)	SR	Study design
Kao, 1994 [16]	J Ped Surg	R2, T2 overlying the 2 rib neck	40	6–16	13.8	66			RCS
Cohen, 1998 [21]	Eur J Surg	T2–T3 ganglia resection	134	6–18		44		98	RCS
Imhof, 1999 [22]	J Ped Surg	T2–T4 two stage	26	11–17	15.4	63	95	57	RCS
Lin, 1999 [23]	J Lap Adv Surg Tech A	below R2–above R3 T2 transection	350	5–17	12.9	86		95	RCS
Lin, 1999 [24]	Ped Surg Int	below R2–above R3 T2	350	5–17	14.2	86		95	RCS
		below R3–above R4, T2–T3	88					89	
Steiner, 2007 [10]	J Ped Surg	T2 ablation R2–R3	35	6–12		70^b^	91	85^c^	RCS
			180	13–18		70^b^	82	86^c^	
Steiner 2008 [25]	Ped Surg Int	T2 ablation R2–R3	116	8–14	13.0	70	91	92	RCS
Buraschi, 2008 [26]	Arch Arg Ped	T2–T3	25	8–18	15.6	28		96^c^	RCS
Wait, 2010 [27]	J Neurosurg Pediatr	T2, R2–R3, over rib heads	54	10–17	15.4y	54	98	98	RCS
Kravarusic, 2012 [28]	Afr J Ped Surg	T2–T3	148		13.8y	38	87	94^c^	RCS
Neves, 2012 [29]	Pediatr Dermatol	T3–T4	30	8–14	13.0	90		77	NCS
Shalaby, 2012 [30]	Ped Surg Int	T2–T3 (*n* = 9) T2–T4 (*n* = 3)	12	6–13	10.1	66		100	PCR
Sinha 2013 [15]	Eur J Ped Surg	T2–T3, R3	44	< 17	12.8	21^d^			RCS
Vialat Soto, 2013 [31]	Rev Cubana Ped	T3–T4, R3–R4	38	11–18	?	24		100	RCS
Bell, 2014 [32]	ANZ J Surg	T2–T4, R2–R4	29	< 19		48		97	RCS
Laje, 2017 [33]	J Ped Surg	T3, R3	28	6–21	14	0	100	100	RCS
Gonzalez López, 2019 [34]	Cirug Pediatr	R3–R4	27	11–19	15.4	15		100	RCS
Current series 2020		T2–T3, R2–R3	98	5–14	12.2	66	99	95	RCS

*R rib* T thoracic level, *CS* compensatory sweating, *OP again* would have procedure performed again, *SR* satisfaction rate (0–100), *RCS* retrospective case series, *NCS* non-randomized comparative study, *PCR* prospectively enrolled case registry

^a^SR for group consisting of children, adolescents, adults

^b^CS for three groups (children, adolescents, adults)

^c^Combination of "very satisfied" and "satisfied"

^d^Reflects only "severe" CS

Compared to the other published series, the cohort evaluated in this study is, on average, the youngest and one of the largest. Therefore, the findings are particularly useful to shed insight into the particularities of treating primary palmar hyperhidrosis by thoracoscopic sympathicolysis in young children. Despite the high rate of postoperative compensatory sweating, children are very satisfied with the procedure and would retrospectively choose to have the intervention performed again. These findings corroborate the findings of other published reports and support the notion that early operative intervention is justified and that there is no rationale to delay treatment for primary palmar hyperhidrosis until adolescence or adulthood.

Our study found that symptoms usually started before school start, and that hyperhidrosis was noticeable by the parents as early as infancy in about a quarter of patients. This implies that primary hyperhidrosis may be a congenital problem, at least in a substantial portion of patients, and that primary care physicians and pediatricians should specifically ask for signs and symptoms during well-child checks and routine care visits. Thereby, early intervention is possible and the associated problems with handwriting and sports participation can be proactively identified and avoided. This supports the recommendations by Steiner et al. [10] who compared children (ages 6–12 years), adolescents (ages 13–18 years), and adults (ages 19–29 years and found that postoperative satisfaction rates were similar, but that the willingness to have undergone the procedure again was significantly higher in the first two groups. Only one report contradicts these findings, arguing that older patients have a greater reduction in sweating compared to younger ones [11]. However, the authors base their conclusions solely on subjective findings.

Sweating patterns are indeed an interesting topic in this context. Normal sweating of the palms and soles is appreciable after birth, while axillary sweating does not begin until puberty [12]. In our series, axillary sweating was never part of the initial complaint, nor a frequent location for compensatory sweating. Therefore, continuing the sympathicolysis as far caudal as thoracic levels T4 or T5 does not seem reasonable in children under 14 years of age. Since there is no universal definition for compensatory sweating, this entity is based on subjective reports by the patients. In our series, it was noted in about two thirds of cases, and only in 6 of these (roughly 10%) had longterm spontaenous resolution. In our patients, the most common areas affected by compensatory sweating were the trunk (36%), back (46%), and soles of the feet (56%). Overall in our patients, we noted that over half had a combination of palmar and plantar hyperhydrosis, even before the procedure. While typically the entity is described as primary palmar hyperhidrosis (PPH), we would argue that children especially may have what should be called primary palmar-plantar hyperhidrosis (PPPH).

Another unexpected finding in our series is the postoperative gustatory sweating that appeared in 6 patients. This is something not previously reported, but was part of our routine follow-up questions since one of our early patients had complained of profuse sweating upon smelling or tasting food. Gustatory sweating is a focal hyperhidrosis that can appear in the context of Frey syndrome [13] after parotid gland surgery or injury. The exact autonomous mechanisms remain unclear [14]. We believe that postoperative gustatory sweating after sympathicolysis or sympathectomy is underreported, since it requires a high level of suspicion by the physician performing the follow-up.

Besides compensatory sweating, complications are exceedingly rare after thoracoscopic sympathicolysis. Although mostly transient Horner syndrome has been described in up to 18% of cases [15], we only encountered one case of mild, but permanent ptosis. There were no intraoperative complications or unusual findings in this particular case. The ptosis was appreciated immediately after the operation. The procedure itself was performed in 2014, so in the latter half of the series, well beyond an expected learning curve. One could speculate that the patient had a variant of a low-lying stellate ganglion. Heat conduction may have played a role, although the technique was performed in exactly the same manner as in the other patients. Over a two year follow-up, by 2016, the ptosis was still appreciable on physical examination, but had improved to the point where her visual axis was not impaired and the patient had no subjective complaints about the ptosis. Other complications such as inadequate resolution or recurrence of hyperhydrosis have been reported in up to 10% of cases [15]. In our series, redo procedures were rarely required (3%).

One of the problems of comparability of studies is that a multitude of different techniques have been used. Not only do different surgeons treat different levels of the sympathetic chain (an overview of the levels is given in Table 2), but different modalities of sympathicolysis or sympathectomy are being employed, ranging from transection, resection, clipping, as well as chemical or CO_2_-laser ablation [16]. In our center, the described technique has produced exceedingly good results, is reproducible and easy to perform. In 2011, an expert consensus was published [17], noting that interruption of the sympathetic chain should be achieved either by electrocautery (as performed in our center) or clipping. Also, disruption just cranial to the third rib produces the highest success rate in treating primary palmar and craniofacial hyperhidrosis. In another systematic review, the authors found that T2-free interventions lowered the risk of Horner's syndrome without compromising efficacy [18]. However, these conclusions are all based on retrospective reports, mainly in the adult population, and should be validated in prospective controlled trials.

Besides its retrospective data acquisition, the main limitation of our study is the non-validated assessment of postoperative satisfaction. Unfortunately, this is common among the other referenced studies listed in our systematic review (Table 2). It makes comparability difficult, although the numbers reported are surprisingly congruent. Overall, the satisfaction rates after the procedure seem to be almost universally high, irrespective of the technique employed or the target levels on the sympathetic chain. Unfortunately, we lost 4 of the 102 patients to follow-up. Several attempts were made to contact these respective patients and families by telephone, mail, and by a search through the social security system. Unfortunately, they had moved away without any follow-up information. The relatively high follow-up quota of 96% (98 of 102 patients) is most likely associated with the geographic circumstances of the Canary islands, in which the population tends to be confined to one island and remains relatively stable and accessible.

In the meantime, a validated quality of life scale, the so-called hidroQOL© tool has been published [19]. Unfortunately, it is currently only available for adults and includes such items as impact on sex life, which is obviously not appropriate for children 14 years and under. We have thus adapted the questionnaire for our pediatric patient population and are currently enrolling patients in a prospective fashion to objectify the impact of the procedure on postoperative quality of life. In one study in adults, quality of life and self-esteem improved significantly after thoracoscopic sympathectomy, despite a high rate (78%) of compensatory sweating [20].

In summary, our technique of thoracoscopic sympathicolysis produced a high degree of patient satisfaction despite a substantial rate of compensatory sweating comparable to other series. More serious complications were exceedingly rare. Considering the excellent outcome, we advocate that hyperhidrosis be treated early in life as soon as it is identified as a problem that impacts on age-appropriate psychosocial development.
